# Identification and Long-Term Detection of *Hepacivirus bovis* Genotype 1 and 2 on a Cattle Farm in Germany

**DOI:** 10.3390/v18010078

**Published:** 2026-01-06

**Authors:** Nadine Hake, Christian von Holtum, Dirk Höper, Ard M. Nijhof, Klaas Dietze, Bernd Hoffmann

**Affiliations:** 1 Tierärztliche Gemeinschaftspraxis Zeven–Selsingen, Bremer Straße 40, 27404 Zeven, Germany; nadine.hake@web.de (N.H.); cvonholtum@gmx.de (C.v.H.); 2 Friedrich-Loeffler-Institut, Südufer 10, 17943 Greifswald-Insel Riems, Germany; dirk.hoeper@fli.de (D.H.); klaas.dietze@fli.de (K.D.); 3 Institute for Parasitology and Tropical Veterinary Medicine, Freie Universität Berlin, Robert-von Ostertag-Str. 7, 14163 Berlin, Germany; ard.nijhof@fu-berlin.de; 4 Veterinary Centre for Resistance Research, Freie Universität Berlin, Robert-von Ostertag-Str. 8, 14163 Berlin, Germany

**Keywords:** *Hepacivirus bovis*, genotype 1, genotype 2, metagenomic analysis, cattle

## Abstract

In 2020, a dairy farm in northwest Germany reported several cows with severe respiratory disease, fever, and reduced milk production. Multiple direct and indirect diagnostic methods were used to identify the cause of the disease. However, the pathogens detected could not be correlated with the severity of the clinical symptoms, so further diagnostic steps were taken. Blood and nasal swab samples were examined using next-generation sequencing (NGS) as part of a metagenomic analysis. For the first time in Germany, *Hepacivirus bovis* genotype 2 was detected. Real-time RT-PCR assays confirmed the presence of BovHepV genotypes 1 and 2 in the herd between 2020 and 2023. Analyses of complete and partial genome sequences demonstrated the presence of different virus variants in the herd over several years. In addition, the sequence data indicated that cattle can be reinfected with viruses belonging either to different BovHepV subtypes or to the same subtype. Although no direct link could be established between the detection of bovine hepaciviruses and the observed clinical symptoms, the PCR and sequence data obtained provide valuable insights into the epidemiology and pathogenesis of BovHepV infections.

## 1. Introduction

Hepatitis C Virus (HCV) and GB virus B were the only members of the genus Hepacivirus within the Flaviviridae family until the discovery of a novel Hepacivirus in dogs in 2011 [1]. In the following years, other Hepacivirus species were found worldwide in different mammals such as horses [2], rodents [3,4], bats [5], cattle [6,7], and donkeys [8], but also in sharks [9] and ducks [10]. The genus Hepacivirus is one of the four genera of the *Flaviviridae* family, and 14 species have been reported to date. The Hepacivirus genome is 9–13 kb in size and is composed of single-stranded, positive-sense and unsegmented RNA. It contains a single long open reading frame (ORF) flanked by 5′- and 3′-terminal non-coding regions and encodes a single polyprotein [11]. The polyprotein is processed proteolytically by signal peptidase as well as the NS2/NS3 and NS3 proteases, resulting in the formation of three structural proteins (Core, E1, and E2) and seven nonstructural proteins (p7, NS2, NS3, NS4A, NS4B, NS5A, and NS5B) [7].

In 2015, independent studies conducted in Ghana [6] and Germany [7] were the first to identify a novel Hepacivirus in cattle, which was subsequently designated *Hepacivirus bovis* (BovHepV). Following these initial reports, numerous detections of BovHepV have been documented worldwide, underscoring its broad geographical distribution [12,13,14,15,16,17,18]. Two different BovHepV-1 subtypes were subsequently found in Germany [19]. BovHepV is classified as a member of the genus Hepacivirus, and strains can be further divided into two different genotypes, of which genotype 1 contains eleven reported subtypes (A-K) [20,21,22]. The subtypes detected in cattle in Germany thus far belonged to subtypes A and F. Based on consistent detection of BovHepV in cattle, they seem to be the natural host. A novel genotype is defined when the viral amino acid (aa) sequence shares less than 77% identity with all previously characterized strains [23]. *Hepacivirus bovis* genotype 2 was first identified in Brazilian cattle in 2019; in the study, the detected nucleotide sequence exhibited 72.6% to 73.8% identity to previously reported NS3 sequences of other BovHepV isolates [24]. One year later, a nucleotide sequence showing 70.8% identity to known NS3 sequences was detected in China [21]. In 2024, BovHepV genotype 2 was detected in bovine blood samples collected in the United States, exhibiting 82.5% nucleotide identity with the Chinese strain IME_01 and 80.5% with the Brazilian strain RS963, indicating that MARC/2019/50 may be a novel subtype within genotype 2. These findings highlight the genetic diversity of *Hepacivirus bovis*. Furthermore, BovHepV has thus far been identified in boas [25], red deer [22], reindeer [26], and sheep [27], indicating that the virus exhibits a broad host range rather than strict host specificity. Further investigations are needed to ascertain the capability of BovHepV to cross species borders and infect other animals [17].

Several studies examined potential associations between BovHepV infection and clinical disease, but no significant correlation could be found [7,17,19]. BovHepV is suspected to be hepatotropic, as the liver and hepatic lymph nodes showed the highest viral loads [7] and viral RNA was recently visualized via fluorescent in situ hybridization in liver cells of an infected cow [17]. However, increased liver enzymes indicative of an acute hepatitis were not observed [7,17]. Ten years after the initial description of *Hepacivirus bovis*, the pathogenesis and transmission routes remain poorly understood. Similar to HCV infection, transmission through infected blood, for instance, through vaccination of livestock without changing needles, has been proposed [7,18]. Regular testing of blood samples from a dairy herd where subtype A circulated for over six months revealed that BovHepV is able to establish chronic infections in cattle, as some animals remained viremic throughout the study period [7]. Nevertheless, many questions regarding the occurrence and clinical significance of bovine hepaciviruses remain unanswered.

In 2020, a dairy farm in Lower Saxony, Germany, reported cases of severe respiratory disease, fever of unknown origin, and decreased milk production in cows, where affected animals did not respond to therapy and either died or had to be euthanized. A metagenomic next-generation sequencing (NGS) approach was employed, which identified *Hepacivirus bovis* (BovHepV), followed by a four-year monitoring period of the infection status of the herd. During this time, cows showed different infection patterns, with some remaining negative, others intermittently testing positive or negative, and a small number staying consistently positive across all years, indicating complex infection dynamics within the herd. The study further documents the first detection of BovHepV genotype 2 in Germany, expanding the known geographic distribution of this genotype and showing an ongoing BovHepV circulation. It suggests the possibility of persistent or recurrent infection with *Hepacivirus bovis* and emphasizes the need for additional research into its pathogenic relevance, transmission pathways, and impact on cattle health.

## 2. Materials and Methods

### 2.1. Diagnostic Sample Collection

As part of the diagnostic examination of the sick cattle at state and private diagnostic laboratories, EDTA blood, nasal swabs, stool samples, milk, and serum samples were taken. In addition, residual serum samples from the annual mandatory government screening program for bovine herpesvirus 1 (BoHV-1) from 2020 to 2023 were used for follow-up investigations.

A definitive cause for the observed respiratory symptoms was not identified, but the outbreak at the dairy farm was self-limiting. To support herd health, gestating and lactating cows were vaccinated against PI3V, BRSV, and *M. haemolytica* in September 2020.

### 2.2. RNA Extraction and Metagenomic Analyses

RNA was extracted from the samples using Trizol LS (ThermoFisher, Darmstadt, Germany) and further processed and sequenced using Ion Torrent S5 on an Ion 530 chip in 400 bp mode, essentially as described in [https://doi.org/10.1038/s41598-018-31496-1 (accessed on 30 August 2018)]. Data analysis was performed using the RIEMS software pipeline [28], and the generated result summaries were manually reviewed to validate the findings. To this end, the reads that were presumed to be of viral origin according to the RIEMS analysis were extracted, and the closest related sequences were searched for again using BLASTn (v2.16.0) in the entire nucleotide sequence database (NCBI nt). In addition, these reads were assembled together with the unclassified reads of the RIEMS analysis, and the resulting contigs (longer continuous sequences composed of several reads) were also analyzed using BLASTn.

### 2.3. Nucleic Acid Extraction and Real-Time RT-PCR

The viral RNA from 100 µL serum was extracted using the NucleoMagVET kit (Macherey-Nagel, Düren, Germany) with the semi-automated KingFisher platform (KingFisher Flex magnetic particle processor, Thermo Fisher Scientific (Waltham, MA, USA)) and eluted in 100 µL elution buffer. To verify successful RNA extraction, an internal control RNA (IC-2 RNA) was added during the extraction process [29]. The RNA was amplified using two newly developed real-time PCR assays for the specific detection of BovHepV genotype 1 and BovHepV genotype 2, modified by an internal control amplification. The final composition of the RT-qPCR reactions was 1.25 µL RNase-free water, 6.25 µL 2× RT-PCR buffer, 0.5 µL RT-PCR enzyme mix, 1 µL BovHepV primer probe mix FAM (10 pmol/µL primer-for, 10 pmol/µL primer-rev, 2.5 pmol/µL FAM probe, in 0.1× Tris-EDTA (pH = 8.0)), 1 µL EGFP mix1-HEX (2.5 pmol/µL primer-for, 2.5 pmol/µL primer-rev, 2.5 pmol/µL HEX probe, in 0.1× Tris-EDTA (pH = 8.0)), and 2.5 µL of template RNA. All RT-qPCRs were performed on the CFX 96 real-time PCR cycler (Bio-Rad, Hercules, CA, USA) using AgPath ID™ One-Step RT-PCR reagents from Applied Biosystems™ (Waltham, MA, USA). The temperature profile used was 10 min at 45 °C, and then 10 min at 95 °C, followed by 45 cycles of 15 s at 95 °C, 30 s at 56 °C, and 30 s at 72 °C. Fluorescence values (FAM, HEX) were recorded during the annealing step. Samples were considered positive if the quantification cycle value (Cq) was <45. Sample processing and analytical procedures followed the approach described by Ries et al. [30], with modifications in primer design and sample material.

Two independent real-time PCR assays were used for this investigation, enabling genotype-specific detection of Hepacivirus RNA. The BovHepV sequences available in GenBank in autumn 2020 and the newly generated BovHepV-2 sequence from the metagenome analysis were used to develop BovHepV genotype 1- and 2-specific real-time PCR assays. The genotype-specific sequences were aligned using MAFFT of the Geneious v.11.1.5 software package (Biomatters, New Zealand) and suitable genome regions were selected. Different combinations of forward primers, reverse primers, and TaqMan probes were tested for sensitivity. The following oligonucleotides were finally used for the BovHepV1-Mix5 assay: bovHepV1-4505-F (5′-CTG CCT ATC CTR AAG GGY GT), bovHepV1-4657-R (5′-GCC ATT CTC ATA RCA RAT CTG C-3′), and bovHepV1-4613-FAM (5′-FAM-ACG TGG CTY AAY GCY GCG CAG AG-BHQ1-3′). For the BovHepV2-Mix4 assay, the following oligonucleotides were combined: BovHepV2-7660-F (5′-CTG GAA CCT ATC CAA ATT YTA TGC-3′), BovHepV2-7763-R (5′-AAC TGG TRG TAA ASA CTC CAG A-3′), and BovHepV2-7722-FAMas (5′-FAM-CCA ATG TAA TTG CCR CGT TGR TCC ACC AT-BHQ1-3′).

Based on the available sequence data for both BoHepV-1 and, in particular, BoHepV-2 at the start of the study in 2020, the corresponding PCR assays were selected and applied. A comprehensive validation of the PCR systems in the sense of a diagnostic PCR was not performed. Rather, the aim was to distinguish between BoHepV-1 and 2 by means of a simple PCR test of serum samples and to obtain a rough overview of the distribution of the genotype in the herd over the years. This pre-screening allowed individual samples to be specifically included in virus genome sequencing. These sequences also confirm the fundamental suitability of PCR methods for detecting BoHepV-1 and BoHepV-2.

### 2.4. Sanger-Sequencing and Sequence Analyses

Suitable primers were derived for sequencing the entire genomes of BovHepV genotype 1 and 2 strains based on previously published sequences and the BovHepV-2 sequence from the metagenome analysis. Using selected primer pairs, overlapping fragments of approximately 600 to 1000 bp were amplified and sequenced. Gaps in the sequences were closed by primer walking. The primer sequences used can be provided upon request.

Two overlapping primer pairs, K6 and K7, were used for partial sequencing of a 1353 bp region from the NS3 gene. The following forward and reverse primers were used for the 752 bp fragment K6: BoHep-3042-F (5′-CGC CGT TAY ATG GGS TTC AC-3′) and BoHep-3793-R (5′-CAC TCA TCA CAR ATR ACC ACA TC-3′). The 926 bp fragment K7 was amplified using primers BoHep-3505-F (5′-CAG TTG GGA RGT ACA RAC TGT-3′) and BoHep-4430-R (5′-CAT TCC RAA CCA MGC CAT ACC-3′).

The SuperScript™ III One-Step RT-PCR System with Platinum™ Taq DNA Polymerase (Thermo Fisher Scientific, Darmstadt, Germany) was used for RT-PCR. In short, 0.75 µL RNase-free water, 6.25 µL 2× reaction mix, 0.5 µL MgSO_4_, 0.5 µL enzyme mixture, 1.0 µL forward primer (10 pmol/µL), and 1.0 µL reverse primer (10 pmol/µL) were mixed, followed by the addition of 2.5 µL template RNA. The RT-PCR was started using the following temperature profile: 30 min at 50 °C, and then 2 min at 95 °C, followed by 50 cycles of 15 s at 95 °C, 30 s at 56 °C, and 60 s at 72 °C. After a final elongation for 5 min at 72 °C, successful amplification of the PCR fragment was verified by electrophoresis. Positive PCR fragments were excised, purified with the QIAquick Gel Extraction Kit (Qiagen, Hilden, Germany), and sequenced with the corresponding forward and reverse primers by Sanger sequencing (Eurofins Genomics, Konstanz, Germany). All primers used were synthesized by Metabion (Planegg, Germany).

The complete and partial BovHepV genome sequences were aligned using MAFFT. A maximum likelihood analysis was subsequently performed using the maximum likelihood method and the Tamura–Nei model, including 1000 bootstrap replicates (Geneious v.11.1.5 software package (Biomatters, New Zealand)).

## 3. Results

### 3.1. Study Background and Farm Structure

From April to August 2020, a dairy farm in Lower Saxony was investigated after several cows developed severe respiratory symptoms, fever of unknown origin, decreased milk production, and increased mortality. Despite treatment with various antibiotics, including amoxicillin, tetracycline, penicillin, and enrofloxacin, as well as painkillers, a total of 24 cows either died peracutely or were euthanized during this period. The farm houses approximately 500 animals across two locations (A and B), which are approx. 2.5 km apart. The stable near the owner’s residence (A) accommodates approx. 250 dairy and dry cows, most of which are Holstein Friesian or Simmental breeds. After birth, calves are housed individually until they are either transferred to a straw pen with an automatic calf feeder or to group igloos. Six- to nine-month-old female calves are routinely transported to the second location (B), where they are served by a bull from the farm’s own stock at an age of 15 to 18 months. The farmer irregularly purchased female Holstein Friesian calves from an age of two weeks and sporadically heifers. Besides milk production and calf rearing, the farm also fattens about 110 bulls on fully slatted floors in stables near the owner’s residence.

To investigate the cause of the observed clinical symptoms, nasal swabs, as well as serum and fecal swabs, were examined for the presence of pathogens. Bacteriological culture of nasal swabs irregularly yielded *Mannheimia haemolytica*, *Klebsiella pneumoniae*, *Acinetobacter baumanii*, *E. coli*, *Proteus* spp., and *Pasteurella multocida*. Antibodies against *Coxiella burnettii*, *Mycoplasma bovis*, Bovine Adenovirus 3, Bovine Coronavirus, Bovine respiratory syncytial virus (BRSV) and Parainfluenza Virus 3 were detected serologically via ELISA. RT-PCR assays for BHV-1, Bovine Adenovirus 3, Bovine Coronavirus, BRSV, PIV 3, Influenza D virus and *C. burnetti* were all negative. PCR tests for the HS-specific genome sequence of *Pasteurella multocida* and *Pasteurella multocida* capsule type B were also negative. The fecal samples were positive for *Clostridium perfringens* but negative for *Salmonella* spp. via culture. Furthermore, a bulk milk sample was positive for antibodies against *C. burnettii* via ELISA. Given that the severity of clinical signs and mortality in the herd were disproportionate to the bacteria and viruses detected, nasal swabs, EDTA blood, and serum samples from six affected cows were submitted to the Friedrich-Loeffler-Institute (FLI). The samples were examined for previously undetected or novel pathogens using a next-generation sequencing (NGS) metagenomic analysis.

### 3.2. Metagenomic Study

Three sample pools (EDTA blood, serum, nasal swabs) were created from the available samples of clinically affected cows and analyzed in a metagenomic analysis. Results showed a high number of reads with approximately 91% sequence identity to bovine hepaciviruses in the blood and serum sample pools (EDTA blood: 252 reads, serum: 6786 reads). From the hepacivirus sequences, the complete genome of a bovine Hepacivirus genotype 2 (accession number in Genbank: OQ979603) with 83.35% identity to Hepacivirus N isolate IME_BovHep_01 (MN691105.1) from China could be determined. No sequences of bovine hepaciviruses were found in the nasal swab pool. Further analysis for viral and bacterial sequences in all three pooled samples yielded only single reads for different pathogen families or unclassified viruses, without any indication that these were involved in the severe symptoms of the disease.

### 3.3. Detection of Bovine Hepacivirus via Real-Time RT-PCR

The detection of bovine hepacivirus genotype 2, previously unknown in Germany, raised the question of whether this virus could be responsible for the clinical symptoms in the herd. After identifying the Hepacivirus, 240 blood samples collected in October 2020 were forwarded to the FLI, where they were tested for BovHepV RNA using genotype 1- and genotype 2-specific real-time RT-PCRs. In addition, serum samples obtained during the annual mandatory government screening program for BoHV-1 in subsequent years (September 2021, October 2022, and October 2023) were also tested using the same real-time RT-PCRs. The aim was to determine the BovHepV infection dynamics over time. The results of the annual herd testing are presented in Table S1 and summarized in Table 1. In 2020, genotype 1 and genotype 2 of *Hepacivirus bovis* were detected in 59 and 27 serum samples, respectively. Six animals tested positive for both BovHepV-1 and BovHepV-2. The prevalence of BovHepV-1 and BovHepV-2 was thus 24.6% (95% confidence interval [CI] 19.2 to 30.6%) and 11.3% (95% CI 7.5 to 16.1%), respectively. Significantly lower cycle of quantification (Cq) values were found for BovHepV-1 compared to BovHepV-2, indicating higher viral genome loads in the BovHepV-1-positive samples. These trends in prevalence and semi-quantitative PCR results were also confirmed over the following three years (Table 1). In each sampling year, BovHepV-1 was detected more frequently (prevalence between 20.1 and 33.7%) than BovHepV-2 (prevalence between 1.1 and 11.2%). Higher viral genome loads were also detectable in BovHepV-1 positive samples. The lowest Cq values for BovHepV-1 ranged between 20.7 and 26.1, whereas those for BovHepV-2 were between 29.3 and 35.3.

Further insights into the infection dynamics can be drawn from the results of the annual herd tests (Table S1), particularly for animals that have been tested over multiple years. A total of 11 animals remained negative for both BovHepV-1 and BovHepV-2 throughout the entire period. Overall, 136 animals tested positive in only a single year, but were negative before and/or after that. Intermittent positivity was observed in 10 animals. A few animals tested positive in all sampling years. Of particular note are cattle R885 and R943, which tested positive for BovHepV-1 PCR in all four tests conducted between 2020 and 2023.

### 3.4. Molecular Characterization of Detected Bovine Hepacivirus 1 and 2

**Figure 1:**
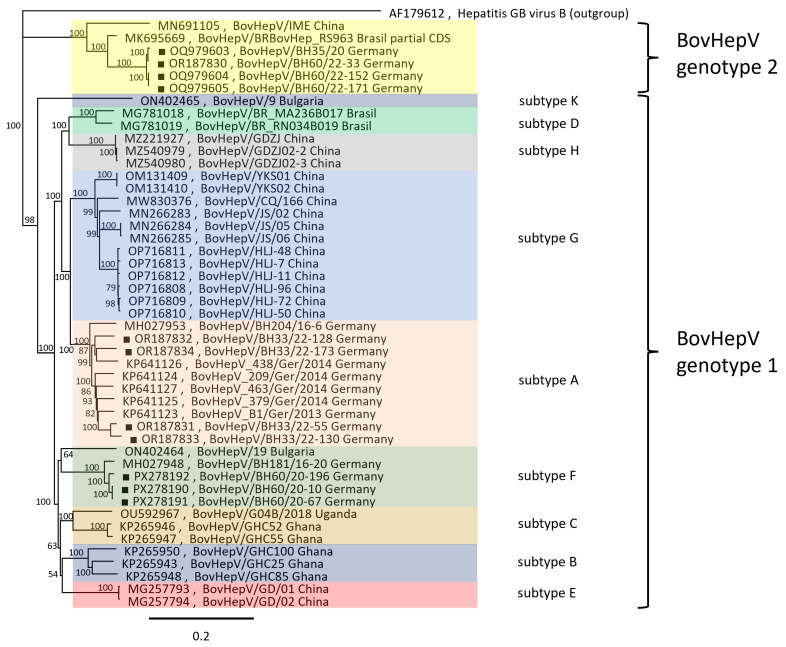
**Phylogenetic analysis of *Hepacivirus bovis* (BovHepV).** The complete BovHepV genome sequences were aligned using MAFFT. Subsequently, a maximum likelihood analysis was performed using the maximum likelihood method and the Tamura–Nei model, including 1000 bootstrap replicates (Geneious v.11.1.5 software package (Biomatters, New Zealand)). The scale indicates the nucleotide substitutions per site. The BovHepV strains analyzed in this study are marked with a black square. The viruses are labeled with their accession number, virus ID, and country of origin. The genome of the hepatitis GB virus was used as an outgroup. The corresponding genotypes and subtypes were defined on the basis of a previous publication [22].

The PCR results raised the question of whether the genomes detected were all from a single strain or from genetically different viruses. For this purpose, a total of seven BovHepV-1 full genomes and three additional BovHepV-2 full genomes (in addition to the previously determined metagenome-based BovHepV-2 genome) were determined using Sanger sequencing. The selection of samples for complete sequencing was based on the determined genome load in the samples, the phylogenetic classification of BoHepV-1 strains based on partial NS3 gene sequences, and the amount of sample still available. Figure 1 shows the phylogenetic tree of the full-length sequences compared to previously published data.

Molecular epidemiological analyses showed that both BovHepV genotypes 1 and 2 circulated in the cattle herd, confirming the real-time RT-PCR analyses. Among the genotype 1 strains, both subtypes 1A and 1F were detected, both of which had previously been reported in samples from Germany. The strains of subtype 1A examined in this study show a sequence identity of 90.2 to 97.0%, which is within the range of previously published subtype 1A strains from Germany. The three full-length genomes of subtype 1F from this study exhibited 98.5 to 99.5% identity to each other and 96.7% identity to the previously published 1F strain from Germany BH181/16-20 (accession no. MH027948). In contrast, the identity between the newly sequenced 1A and 1F strains is only 79.6 to 83.5%.

Genotype 2 of BovHepV has not previously been detected in Germany. The four full-length BovHepV-2 genomes sequenced from the herd showed 99.1 to 99.9% nucleotide identity to each other, indicating a close genetic relationship with some sequence variation. The nucleotide identity of the German BovHepV-2 strains to the prototype genotype 2 virus IME from China (accession number MN691105) is 82.8–89.0%. Finally, the nucleotide identity between German BovHepV genotypes 1 and 2 was approx. 67%, confirming their genetic distinction.

The sequence data of the complete virus sequences were checked for the presence of a long open reading frame (ORF) representing the genetic information for a corresponding polyprotein. No further studies on the proteolytic cleavage of the polyprotein and the viral individual proteins derived from it have been conducted. Any statements on this would be pure speculation and cannot be substantiated by our own investigations. For this reason, only the sequences were determined, and no further annotation of possible individual proteins was conducted. No evidence of recombination between BoHepV genotype 1 and 2 strains was found.

**Figure 2:**
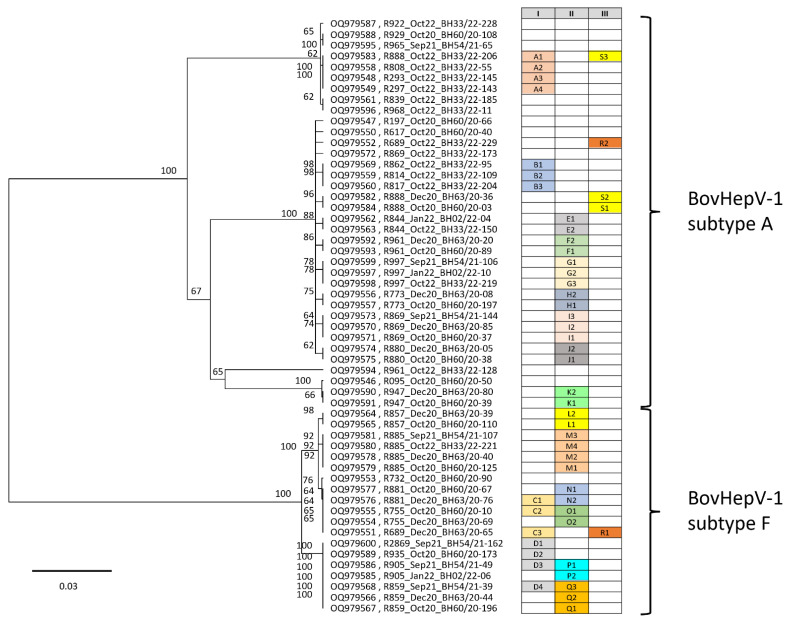
**Phylogenetic analysis of 55 partial BovHepV-1 sequences of the NS3 gene from the years 2020–2022.** The partial BovHepV genome sequences were aligned using MAFFT. Subsequently, a maximum likelihood analysis was performed using the maximum likelihood method and the Tamura–Nei model, including 1000 bootstrap replicates. The scale indicates the nucleotide substitutions per site. The viruses are labeled with their accession number, cattle ID, sampling date, and sample ID. The table on the right summarizes several key observations: (I) different cattle carry genetically identical virus, (II) identical viruses persist for months or years in the same animal, (III) reinfections with novel BovHepV variants, either with alternative subtypes or genetically distinct strains within the same subtype, are possible. The letter–number combination continuously identifies the corresponding animals/samples in a subgroup. Examples of how to interpret the table in the figure are as follows: Column I: The four cattle in subgroup A (A1, A2, A3, and A4) all carry a virus with the identical NS3 gene sequence. Column II: In cattle R885, the identical virus sequence was found in four samples taken between October 2020 and 22 October (M1 to M4 in column II). The accession numbers and sample IDs are also summarized in Supplemental Materials (Table S2).

As shown in Table 1 and Supplementary Table S1, numerous BovHepV-1 positive samples, some with high viral genome load, were detected by RT-qPCR between 2020 and 2023. In some cases, animals tested positive by PCR over two or more consecutive years. This raised the question of whether, in addition to subtypes 1A and 1F, other BovHepV-1 subtypes were circulating in the herd and how closely related the detected strains were. In addition, the sequencing of samples from cattle that tested positive multiple times was intended to clarify whether these animals always harbored the same virus strain or were repeatedly infected with genetically distinct variants. For this purpose, partial sequencing of a 1353 bp fragment of the NS3 gene was performed on high-load BovHepV-1 samples collected between 2020 and 2022. A total of 55 sequences from 31 cattle were analyzed. The phylogenetic relationships of these NS3 sequences are shown in Figure 2.

Analysis of the partial NS3 sequences confirmed that only subtypes 1A and 1F were present in the population, and no evidence for the presence of other subtypes was found. As expected from the full-length genome sequencing data, there was not just one virus variant within the subtype, but a whole range of similar, but not identical, virus sequences. On closer analysis, the subtype 1A strains could be divided into four subclusters with significant sequence differences of approximately 90 to 120 nucleotides. One example is the single-strain R961_Oct20_BH33/22-128 (acc. no. OQ979594), which has nucleotide differences of 88 and 89 nucleotides, respectively, compared to the next most closely related strains of the subcluster below (acc. nos. OQ979546, OQ979590, OQ979591). Different sequences can also be found in subtype 1F strains, although the variations are far less pronounced. Within subtype 1F, a maximum of 20 nucleotides differ in the sequenced NS3 region.

On the other hand, however, the data also clearly show that different cattle carry the identical virus. In the table in Figure 2, these animals are listed under Column (I) in alphabetical order with a letter and number. Column (II) groups sequences proving that the identical virus persists in one and the same animal for months or even years. Particularly striking here is animal R885, from which sequences could be generated in 2020, 2021, and 2022, and all of these sequences were completely identical (M1–M4). The sequence data summarized in column (III) are particularly interesting. This data clearly shows that a cow infected with subtype 1F can also be reinfected with another strain of subtype 1A (R1 = cattle R689_Sep21 and R2 = cattle R689_Oct22). Even more interesting are the sequences of cow R888; in this cow, the identical virus was detected in October and December 2020 (S1 and S2), and in October 2022 (S3), a significantly different sequence was determined. The difference between the S1/S2 sequences and the S3 sequence is 115 nucleotides.

## 4. Discussion

Despite extensive diagnostics for a wide variety of bacteria and viruses, no pathogen could be found in a clinically abnormal dairy herd that would have provided a plausible explanation for the severe clinical symptoms. Additional metagenome analysis identified *Hepacivirus bovis* in the blood of sick cattle. Extensive PCR testing and virus sequencing showed that two subtypes (1A and 1F) of BovHepV genotype 1 were circulating in the herd. In addition, BovHepV genotype 2 was detected for the first time in Germany. The degree of similarity in nucleotide identity among the German BoHepV-2 strains falls within the range that, as for BovHepV-1, would facilitate subtyping. However, given the limited number of BovHepV-2 genome sequences currently publicly available, defining new subtypes at this stage does not appear justified. Both genotypes with different virus variants circulate in the herd, in some cases in individual animals, over several years. Since both clinically symptomatic and asymptomatic cows in our herd were infected with BovHepV and, in contrast, no BovHepV was detected in some clinically symptomatic animals, a direct link between clinical symptoms and infection with *Hepacivirus bovis* cannot be established. Various other studies also found no clear link between BovHepV infection and clinical symptoms [7,17,19], although hepatotropism was detected [7].

In contrast, research on *Hepacivirus equi* (EquHepV) is more advanced. The highest viral loads were found in the liver [31,32] and spleen [32], with the localization of viral RNA in hepatocytes confirmed by fluorescent in situ hybridization [33]. EquHepV can cause acute infections with an increase in liver-specific enzyme activities or progress to chronic infection, with enzyme levels remaining within or slightly above the reference range [32]. The studies also showed that most horses undergo seroconversion and clear the viral infection or show seroconversion without detection of viral RNA. Some horses were unable to eliminate EquHepV infection despite an increase in antibodies. In the present study, 19 of 403 cows tested positive for *Hepacivirus bovis* in at least two consecutive years, suggesting possible persistence. In addition, five animals tested alternately positive and negative during the three-year testing period. On the one hand, this may indicate reinfection with the virus, but it may also be due to a decrease in viral load below the PCR detection limit. However, the documented reinfections with BovHepV-1 of a different subtype, but also within the same subtype, clearly show that reinfections are possible in principle. It can therefore be assumed that the immune response of cattle to infection with BovHepV is not very pronounced or is lost within a very short time.

Persistent infections lasting seven to 20 months have been described for EquHepV [31,32,34,35,36]. Retrospectively, one study described a persistence of 12 years [31]. Reinfection in horses has not been described to date. The symptoms of EquHepV infection range from subclinical hepatitis with no or only a slight increase in liver enzymes [31,37] to hepatitis with jaundice and liver failure [38] and hepatocyte necrosis [39] to chronic infections that can last for several years [31,37], depending on age, breed, and immune status. Since horses generally live significantly longer than cattle, it is possible that liver diseases caused by *Hepacivirus bovis* do not manifest themselves in cattle. Long-term studies on cattle persistently infected with BovHepV must show in the future whether this hypothesis can be scientifically substantiated.

Since the first description of *Hepacivirus bovis* in 2015 [6,7], various studies have shown its worldwide distribution and great genetic diversity. *Hepacivirus bovis* is 1 of 14 recognized Hepacivirus species and primarily infects cattle [23]. It is divided into two genotypes: genotype 1 with subtypes A-K and genotype 2. Subtypes of genotype 1 have been identified in several countries, including Brazil [12,40], Bulgaria [22], China [14,20,41], Germany [7,17,19], Ghana [6], and Italy [18], while genotype 2 has been found in Brazil [24], China [21], the Czech Republic [22], and the USA [42]. To the authors’ knowledge, this study represents the first detection of genotype 2 of *Hepacivirus bovis* in Germany. Furthermore, it is the first detection of co-infection with genotypes 1 and 2 in cows. To date, only the circulation of two subtypes of genotype 1 in one animal has been described, but not the circulation of both genotypes [22]. Further studies are needed to show whether the qualitative and semi-quantitative differences in PCR detection of BovHepV-1 and 2 found in the dairy herd are also observed in other cattle herds. The BovHepV-1- and BovHepV-2-specific PCR assays established in this study appear to be suitable methods for this purpose. Various PCR methods for the detection of BovHepV have been published (references), but to our knowledge, none of them can specifically distinguish between genotype 1 and 2.

## 5. Conclusions

In conclusion, this case report revealed ongoing infection dynamics of BovHepV on a dairy farm, providing evidence of possible persistent or reinfection with *Hepacivirus bovis*. In addition, it documents the first detection of *Hepacivirus bovis* genotype 2 and the co-circulation of genotype 1 and 2 in Germany. Further studies are needed to elucidate the virus’s pathogenesis, transmission routes, and potential effects on infected cows.

## Figures and Tables

**Table 1:** Summary of the real-time PCR detection of *Hepacivirus bovis* on serum samples based on annual herd bleeding.

	October 2020		September 2021		October 2022		October 2023	
	BovHepV-1	BovHepV-2	BovHepV-1	BovHepV-2	BovHepV-1	BovHepV-2	BovHepV-1	BovHepV-2
tested positive/ total number of samples	59/240	27/240	53/264	3/264	72/245	7/245	83/246	9/246
prevalence (%)	24.58	11.25	20.08	1.14	29.39	2.86	33.74	3.66
95% confidence interval (%)	19.2–30.6	7.5–16.1	15.7–25.8	0.2–3.3	23.7–35.6	1.1–5.9	27.8–40.0	1.7–6.9
highest virus load (=low Cq)	25.7	29.3	26.1	35.3	23.8	32.2	20.7	32.0
lowest virus load (=high Cq)	36.8	40.4	39.0	42.9	38.7	36.3	40.4	44.2
co-infections with both genotypes	6		0		0		2	

## Data Availability

The data presented in this study are available in the manuscript, the Supplementary Materials, or on request from the corresponding author.

## Supplementary Materials

The following supporting information can be downloaded at https://www.mdpi.com/article/10.3390/v18010078/s1, Table S1: Results of real-time RT-PCR for BovHepV-1 and BovHepV-2 for 2020 to 2023; Table S2: List of accession numbers and corresponding sample ID for the sequences analyzed in Figure 2.
