# Supplementary material for: Identification and Long-Term Detection of Hepacivirus bovis Genotype 1 and 2 on a Cattle Farm in Germany
Source: Viruses. 2026 Jan 6;18(1):78. doi: 10.3390/v18010078 (PMC12846713; doi:10.3390/v18010078)
Supplement: Supplementary file 1 [file viruses-18-00078-s001.zip › viruses-4016936-supplementary.pdf]

Table S1: Results of the real-time RT-PCR for BovHepV-1 and BovHepV-2 for the years 2020 to 2023

Positive results in the serum samples were listed in red and the according Cq value. Negative results were presented in green and “no Cq”. If no serum was available for testing, the corresponding well remained empty.

	Oct 2020		Sep 2021		Oct 2022		Oct 2023	
Cattle-ID	BovHep1 Mix 5	BovHep2 Mix 4	BovHep1 Mix 5	BovHep2 Mix 4	BovHep1 Mix 5	BovHep2 Mix 4	BovHep1 Mix 5	BovHep2 Mix 4
R719	no Cq	no Cq						
R955	no Cq	no Cq	34.38	no Cq				
R888	28.18	no Cq	no Cq	no Cq	no Cq	no Cq	no Cq	no Cq
R092	no Cq	35.34	no Cq	no Cq				
R930	no Cq	no Cq						
R878	30.65	33.02						
R924	30.74	no Cq			35.91	no Cq		
R962	no Cq	no Cq	no Cq	no Cq				
R905	32.37	no Cq						
R755	28.75	no Cq	no Cq	no Cq	no Cq	no Cq		
R958	no Cq	no Cq	no Cq	no Cq	28.42	no Cq		
R933	32.7	no Cq	no Cq	no Cq	37.30	no Cq		
R901	no Cq	no Cq	no Cq	no Cq	no Cq	no Cq	no Cq	no Cq
R094	no Cq	33.28	33.44	no Cq	29.95	no Cq	no Cq	no Cq
R740	no Cq	no Cq	31.55	no Cq	no Cq	no Cq	no Cq	no Cq
R726	no Cq	no Cq						
R966	no Cq	no Cq	no Cq	no Cq				
R883	29.01	no Cq	no Cq	no Cq				
R892	no Cq	no Cq	no Cq	no Cq	no Cq	no Cq	33.71	no Cq
R968	no Cq	no Cq	no Cq	no Cq	26.76	no Cq		
R999	no Cq	no Cq	no Cq	no Cq	no Cq	no Cq	no Cq	no Cq
R793	no Cq	no Cq	no Cq	no Cq	no Cq	no Cq	no Cq	no Cq
R097	no Cq	no Cq	36.16	no Cq	30.34	no Cq		
R417	no Cq	no Cq	no Cq	no Cq				
R098	no Cq	no Cq			31.17	no Cq	no Cq	no Cq
R872	no Cq	34.27						
R860	no Cq	36.28	no Cq	no Cq	30.01	no Cq	no Cq	no Cq
R922	no Cq	no Cq						
R766	no Cq	35.25						
R902	no Cq	no Cq	37.10	no Cq	32.03	no Cq		
R705	32.26	no Cq						
R914	no Cq	no Cq	33.84	no Cq				
R727	no Cq	29.27						
R945	no Cq	no Cq	31.34	no Cq				
R996	no Cq	no Cq	no Cq	no Cq				
R995	no Cq	no Cq	no Cq	no Cq				
R869	29.09	no Cq	33.49	no Cq	27.70	no Cq		
R880	28.29	no Cq	no Cq	no Cq	no Cq	no Cq		
R947	28.77	no Cq	no Cq	no Cq	no Cq	no Cq	32	no Cq
R617	28.82	no Cq	no Cq	no Cq	no Cq	no Cq		
R690	no Cq	no Cq						
R923	no Cq	no Cq	no Cq	no Cq	no Cq	no Cq		
R927	no Cq	no Cq	no Cq	no Cq				
R947	no Cq	no Cq	no Cq	no Cq				
R920	no Cq	no Cq	31.27	no Cq				
R267	no Cq	no Cq	37.78	no Cq	no Cq	no Cq	no Cq	no Cq
R923	35.76	35.13	no Cq	no Cq	no Cq	no Cq		

R777	no Cq	no Cq	no Cq	no Cq	no Cq	no Cq		
R736	32.02	no Cq	no Cq	no Cq	no Cq	no Cq	23.44	no Cq
R095	27.76	no Cq	no Cq	no Cq	no Cq	no Cq	no Cq	no Cq
R731	no Cq	no Cq	33.76	no Cq	no Cq	no Cq		
R421	31.04	34.85	no Cq	no Cq	30.78	no Cq		
R945	no Cq	no Cq	31.77	no Cq	no Cq	no Cq	no Cq	no Cq
R921	no Cq	no Cq	no Cq	no Cq	no Cq	no Cq	30.67	no Cq
R257	no Cq	no Cq						
R792	no Cq	no Cq	34.15	no Cq				
R919	no Cq	no Cq						
R952	no Cq	no Cq	no Cq	no Cq	no Cq	no Cq	34.87	no Cq
R281	29.01	no Cq	32.36	no Cq	no Cq	no Cq	no Cq	no Cq
R935	no Cq	no Cq						
R759	no Cq	no Cq	no Cq	no Cq	no Cq	no Cq		
R768	32.47	no Cq	37.69	no Cq				
R944	no Cq	no Cq	no Cq	no Cq	30.76	no Cq		
R730	33.43	no Cq						
R064	no Cq	no Cq						
R197	27.57	no Cq						
R881	26.7	no Cq	no Cq	no Cq	no Cq	no Cq		
R082	no Cq	no Cq	39.00	no Cq	no Cq	no Cq		
R931	no Cq	no Cq	no Cq	no Cq				
R726	no Cq	no Cq	no Cq	no Cq	32.79	no Cq	39.63	no Cq
R907	no Cq	33.09	no Cq	no Cq	no Cq	no Cq	no Cq	no Cq
R770	30.98	no Cq	no Cq	no Cq				
R961	no Cq	no Cq	no Cq	no Cq	24.25	no Cq	28.49	no Cq
R962	no Cq	no Cq						
R061	no Cq	no Cq						
R939	no Cq	no Cq						
R950	29.31	no Cq	no Cq	no Cq				
R988	no Cq	no Cq	no Cq	no Cq				
R691	no Cq	no Cq	no Cq	no Cq				
R865	no Cq	no Cq	34.09	no Cq	no Cq	no Cq	no Cq	no Cq
R854	no Cq	no Cq	no Cq	35.27				
R674	no Cq	no Cq						
R696	no Cq	no Cq						
R665	no Cq	no Cq	no Cq	no Cq	no Cq	no Cq		
R929	no Cq	no Cq	no Cq	no Cq	no Cq	no Cq	no Cq	no Cq
R698	no Cq	no Cq						
R728	35.67	35.07						
R293	31.7	no Cq	no Cq	no Cq	no Cq	no Cq	no Cq	37.77
R961	28.28	no Cq						
R732	27.1	no Cq	no Cq	no Cq				
R715	no Cq	35.71	35.34	no Cq	no Cq	no Cq		
R739	no Cq	34.45	no Cq	no Cq				
R943	31.48	no Cq	35.90	no Cq	27.00	no Cq	29.33	no Cq
R905	no Cq	no Cq	29.89	no Cq	32.27	no Cq		
R939	no Cq	no Cq	no Cq	no Cq	no Cq	no Cq	no Cq	no Cq
R882	no Cq	no Cq	no Cq	no Cq	30.63	no Cq	35.57	32.22
R627	36.22	no Cq						
R100	no Cq	no Cq	no Cq	no Cq	no Cq	no Cq	no Cq	no Cq
R923	no Cq	no Cq	32.71	no Cq	no Cq	no Cq	no Cq	no Cq
R948	no Cq	no Cq	32.89	no Cq	no Cq	no Cq	no Cq	no Cq
R957	no Cq	no Cq						

R953	no Cq	no Cq						
R965	no Cq	no Cq	29.03	no Cq	26.56	no Cq		
R944	no Cq	no Cq	34.29	no Cq				
R703	32.23	no Cq	no Cq	no Cq				
R941	no Cq	no Cq	no Cq	no Cq	33.05	no Cq	no Cq	no Cq
R619	36.81	no Cq						
R929	28.25	no Cq	no Cq	no Cq	no Cq	no Cq		
R480	no Cq	no Cq	no Cq	no Cq				
R857	29.04	no Cq	no Cq	no Cq				
R946	no Cq	no Cq	no Cq	no Cq	30.17	no Cq	30.79	no Cq
R932	no Cq	no Cq						
R928	no Cq	no Cq						
R933	no Cq	no Cq						
R689	no Cq	no Cq	no Cq	no Cq	29.46	no Cq	no Cq	no Cq
R539	no Cq	36.27	no Cq	no Cq	no Cq	no Cq	no Cq	no Cq
R870	no Cq	no Cq	35.18	no Cq	no Cq	no Cq	no Cq	no Cq
R889	34.03	no Cq						
R719	34.62	no Cq						
R659	no Cq	no Cq						
R740	29.48	no Cq	no Cq	no Cq	no Cq	no Cq		
R953	28.49	no Cq						
R917	no Cq	no Cq						
R876	no Cq	no Cq			no Cq	no Cq	no Cq	no Cq
R885	27.28	no Cq	31.41	no Cq	27.35	no Cq	28.93	no Cq
R728	no Cq	no Cq						
R744	no Cq	no Cq	no Cq	no Cq	no Cq	no Cq	no Cq	35.25
R780	no Cq	no Cq						
R956	no Cq	no Cq						
R918	no Cq	no Cq	33.47	no Cq	no Cq	no Cq	no Cq	no Cq
R930	no Cq	no Cq						
R884	no Cq	no Cq						
R623	no Cq	no Cq	no Cq	no Cq	no Cq	no Cq	40.39	no Cq
R754	29.57	34.42						
R940	no Cq	no Cq	no Cq	35.73				
R926	no Cq	no Cq	no Cq	no Cq				
R783	30.27	39.05	no Cq	no Cq				
R887	30.03	no Cq						
R938	no Cq	no Cq	34.62	no Cq				
R958	no Cq	no Cq	no Cq	no Cq	no Cq	no Cq	30.82	no Cq
R925	no Cq	no Cq	no Cq	no Cq				
R868	no Cq	no Cq	36.72	no Cq	30.82	no Cq		
R960	no Cq	no Cq	no Cq	42.86	29.23	no Cq		
R263	no Cq	no Cq	no Cq	no Cq	32.61	no Cq		
R288	31.59	no Cq						
R727	35.05	no Cq						
R771	no Cq	no Cq	no Cq	no Cq	no Cq	no Cq	no Cq	no Cq
R949	no Cq	no Cq	no Cq	no Cq	31.42	no Cq	no Cq	no Cq
R664	no Cq	no Cq	no Cq	no Cq				
R660	36.17	no Cq	no Cq	no Cq				
R963	no Cq	no Cq	no Cq	no Cq				
R900	no Cq	31.44	no Cq	no Cq	31.90	no Cq	no Cq	no Cq
R936	no Cq	no Cq	no Cq	no Cq	no Cq	no Cq		
R959	no Cq	no Cq	no Cq	no Cq	37.07	no Cq	no Cq	no Cq
R966	no Cq	no Cq	no Cq	no Cq	no Cq	no Cq	30.25	no Cq

R950	no Cq	no Cq	no Cq	no Cq	no Cq	no Cq		
R772	no Cq	no Cq	no Cq	no Cq				
R637	no Cq	no Cq	no Cq	no Cq				
R529	no Cq	no Cq	no Cq	no Cq				
R916	no Cq	no Cq	32.10	no Cq	no Cq	no Cq		
R267	no Cq	no Cq			no Cq	no Cq	no Cq	no Cq
R091	no Cq	36.52	no Cq	no Cq				
R898	no Cq	no Cq	33.51	no Cq				
R790	no Cq	no Cq	no Cq	no Cq				
R751	29.47	no Cq	no Cq	no Cq	no Cq	no Cq		
R533	no Cq	no Cq	no Cq	no Cq				
R951	no Cq	no Cq						
R000	no Cq	no Cq	34.62	no Cq	33.43	no Cq	34.03	no Cq
R748	no Cq	no Cq	no Cq	no Cq				
R938	no Cq	no Cq	30.36	no Cq	no Cq	no Cq	no Cq	no Cq
R890	no Cq	31.13	no Cq	no Cq	no Cq	no Cq	no Cq	no Cq
R864	no Cq	no Cq	no Cq	no Cq	no Cq	no Cq	no Cq	no Cq
R935	25.69	no Cq	no Cq	no Cq				
R682	no Cq	no Cq	no Cq	no Cq				
R259	no Cq	no Cq	no Cq	no Cq	no Cq	no Cq	31.05	no Cq
R932	no Cq	no Cq	38.47	no Cq	33.08	no Cq		
R400	no Cq	no Cq						
R970	no Cq	no Cq	35.60	no Cq				
R514	no Cq	40.4						
R769	no Cq	32.88						
R969	no Cq	no Cq	no Cq	no Cq				
R970	no Cq	no Cq	no Cq	no Cq	no Cq	no Cq	no Cq	no Cq
R678	no Cq	no Cq						
R992	no Cq	no Cq	no Cq	no Cq	31.10	no Cq		
R901	no Cq	no Cq	no Cq	no Cq	29.31	no Cq	no Cq	no Cq
R925	31.78	no Cq	no Cq	no Cq				
R910	no Cq	no Cq						
R791	no Cq	no Cq						
R998	no Cq	no Cq	35.61	no Cq	26.51	no Cq		
R622	no Cq	35.96	no Cq	no Cq	no Cq	no Cq		
R852	28.06	no Cq	no Cq	no Cq	35.55	no Cq		
R105	no Cq	no Cq	no Cq	no Cq	no Cq	no Cq	no Cq	no Cq
R736	no Cq	no Cq	no Cq	no Cq	no Cq	no Cq	31.74	no Cq
R931	no Cq	no Cq	no Cq	no Cq	no Cq	no Cq		
R926	no Cq	40.41						
R859	28.5	no Cq	29.93	no Cq				
R773	28.25	no Cq	no Cq	no Cq				
R861	no Cq	35.86	no Cq	no Cq				
R518	no Cq	no Cq						
R796	29.26	no Cq	no Cq	no Cq	no Cq	no Cq	28.64	no Cq
R266	no Cq	no Cq	no Cq	no Cq	33.78	no Cq	no Cq	no Cq
R261	no Cq	no Cq	32.39	no Cq	no Cq	no Cq		
R707	no Cq	no Cq	no Cq	no Cq				
R736	no Cq	no Cq						
R028	no Cq	33.54	36.12	no Cq	no Cq	no Cq		
R867	30.74	no Cq	no Cq	no Cq	no Cq	no Cq		
R729	30.09	no Cq						
R874	no Cq	no Cq						
R909	no Cq	no Cq						

R910	no Cq	no Cq						
R967	no Cq	no Cq	no Cq	no Cq	no Cq	33.06		
R670	32.22	no Cq						
R314	33.55	no Cq	no Cq	no Cq	no Cq	no Cq	no Cq	no Cq
R997	no Cq	no Cq	no Cq	no Cq				
R866	no Cq	39.25	no Cq	no Cq	no Cq	no Cq		
R680	no Cq	no Cq						
R919	no Cq	no Cq	no Cq	no Cq	no Cq	no Cq	no Cq	no Cq
R937	no Cq	no Cq	no Cq	no Cq	no Cq	no Cq	no Cq	no Cq
R896	32.02	no Cq	no Cq	no Cq				
R913	no Cq	no Cq	31.22	no Cq	no Cq	no Cq	no Cq	no Cq
R954	no Cq	no Cq	no Cq	no Cq				
R943	no Cq	no Cq	30.24	no Cq				
R779	no Cq	no Cq	no Cq	no Cq				
R659	no Cq	no Cq						
R698	33.73	no Cq						
R968	no Cq	no Cq						
R964	no Cq	no Cq	no Cq	no Cq				
R942	no Cq	no Cq	34.11	no Cq	no Cq	no Cq		
R265	no Cq	no Cq	no Cq	no Cq	no Cq	no Cq	no Cq	no Cq
R260	no Cq	no Cq						
R659	no Cq	no Cq	no Cq	no Cq				
R844	30.02	no Cq	32.24	no Cq	27.83	no Cq		
R937	no Cq	no Cq	no Cq	no Cq	no Cq	no Cq	26.02	no Cq
R851	no Cq	no Cq	no Cq	no Cq	27.28	no Cq	29.24	no Cq
R196	no Cq	no Cq						
R430	no Cq	no Cq	38.07	no Cq			no Cq	no Cq
R167	30.5	no Cq						
R264	no Cq	no Cq						
R951	no Cq	no Cq	32.93	no Cq	35.30	no Cq	no Cq	no Cq
R775	no Cq	35.48						
R098			33.45	no Cq				
R269			34.74	no Cq				
R997			29.92	no Cq	27.93	no Cq		
R930			31.05	no Cq				
R922			35.78	no Cq	38.70	no Cq		
R2869			26.10	no Cq	no Cq	no Cq	no Cq	no Cq
R257			27.28	no Cq				
R801			no Cq	no Cq	no Cq	no Cq	28.68	no Cq
R293			no Cq	no Cq	26.40	no Cq	36.83	no Cq
R840			no Cq	no Cq				
R15848			no Cq	no Cq	no Cq	no Cq	27.4	no Cq
R15832			no Cq	no Cq	no Cq	no Cq	34.31	no Cq
R83933			no Cq	no Cq	no Cq	no Cq		
R83884			31.54	no Cq	no Cq	36.02		
R83876			no Cq	no Cq				
R15854			no Cq	no Cq	no Cq	no Cq		
R32878			no Cq	no Cq	no Cq	no Cq	no Cq	no Cq
R15856			no Cq	no Cq	29.49	no Cq		
R15847			no Cq	no Cq	no Cq	no Cq	no Cq	no Cq
R83977			no Cq	no Cq				
R02953			no Cq	no Cq				
R83995			no Cq	no Cq	29.20	no Cq	32.41	no Cq
R83984			no Cq	no Cq	31.21	no Cq	no Cq	no Cq

R83983			no Cq	no Cq	no Cq	no Cq	no Cq	no Cq
R15839			no Cq	no Cq	26.34	no Cq		
R15838			no Cq	no Cq				
R02924			no Cq	no Cq			no Cq	no Cq
R15851			no Cq	no Cq	no Cq	no Cq	no Cq	no Cq
R32876			no Cq	no Cq	no Cq	no Cq	no Cq	no Cq
R15833			no Cq	no Cq	no Cq	no Cq	no Cq	32.16
R15835			no Cq	no Cq				
R32888			no Cq	no Cq	26.86	no Cq	35.8	no Cq
R15804			no Cq	no Cq	no Cq	no Cq		
R15845			no Cq	no Cq				
R32893			no Cq	no Cq	no Cq	no Cq	39.37	no Cq
R32866			no Cq	no Cq	29.29	no Cq	no Cq	32.03
R15853			no Cq	no Cq	no Cq	no Cq	no Cq	no Cq
R15860			no Cq	no Cq	no Cq	no Cq	30.03	no Cq
R27296			no Cq	no Cq	31.43	no Cq	no Cq	no Cq
R27297			no Cq	no Cq	25.33	no Cq	no Cq	no Cq
R83996			no Cq	no Cq	no Cq	35.12	39.61	no Cq
R32889			no Cq	no Cq				
R15837			no Cq	no Cq	no Cq	32.21	no Cq	34.75
R15803			no Cq	no Cq	no Cq	no Cq	28.72	no Cq
R32884			no Cq	no Cq				
R83999			no Cq	no Cq	no Cq	no Cq	no Cq	no Cq
R32862			no Cq	no Cq	26.53	no Cq	no Cq	no Cq
R32874			no Cq	no Cq				
R15857			no Cq	no Cq	no Cq	no Cq	35.85	no Cq
R83878			no Cq	no Cq	no Cq	no Cq	no Cq	no Cq
R83974			no Cq	no Cq	no Cq	no Cq	no Cq	no Cq
R83973			no Cq	no Cq	no Cq	no Cq	no Cq	no Cq
R19751			no Cq	no Cq	no Cq	no Cq	no Cq	no Cq
R83990			no Cq	no Cq				
R86673			no Cq	no Cq	no Cq	no Cq	no Cq	no Cq
R04726			no Cq	no Cq				
R83991			no Cq	no Cq	27.50	no Cq	no Cq	no Cq
R83988			no Cq	no Cq	no Cq	no Cq	no Cq	no Cq
R32871			no Cq	no Cq				
R15858			no Cq	no Cq	no Cq	no Cq	29	no Cq
R83981			no Cq	no Cq	no Cq	33.55	35.47	44.21
R15841			no Cq	no Cq	29.15	no Cq		
R15850			no Cq	no Cq	no Cq	no Cq	no Cq	no Cq
R83972			no Cq	no Cq	no Cq	no Cq	no Cq	no Cq
R83975			no Cq	no Cq				
R32894			no Cq	no Cq	no Cq	no Cq	29.62	no Cq
R33727			no Cq	no Cq	33.02	no Cq		
R15805			no Cq	no Cq				
R15846			no Cq	no Cq	30.31	no Cq	no Cq	33.03
R32879			no Cq	no Cq	no Cq	no Cq	no Cq	no Cq
R83978			no Cq	no Cq	31.21	no Cq	34.18	no Cq
R83976			no Cq	no Cq	29.46	no Cq		
R42260			no Cq	no Cq				
R15843			no Cq	no Cq	34.12	no Cq	32.56	no Cq
R32881			no Cq	no Cq				
R32863			no Cq	no Cq	no Cq	34.39		
R84000			no Cq	no Cq	no Cq	no Cq		

R83989			no Cq	no Cq	29.34	no Cq	no Cq	no Cq
R15852			no Cq	no Cq	27.33	no Cq		
R15855			no Cq	no Cq	no Cq	no Cq	no Cq	no Cq
R15834			no Cq	no Cq	no Cq	36.27	no Cq	31.96
R15807			no Cq	no Cq	no Cq	no Cq	no Cq	no Cq
R15802			no Cq	no Cq	31.39	no Cq	29.06	no Cq
R15809			no Cq	no Cq	no Cq	no Cq	no Cq	no Cq
R15831			no Cq	no Cq	no Cq	no Cq	32.65	no Cq
R32901			no Cq	no Cq				
R00288			no Cq	no Cq				
R83971			no Cq	no Cq	no Cq	no Cq	29.98	no Cq
R83985			no Cq	no Cq				
R83993			no Cq	no Cq				
R83998			no Cq	no Cq	no Cq	no Cq	no Cq	no Cq
R32864			no Cq	no Cq			no Cq	no Cq
R32875			no Cq	no Cq	no Cq	no Cq		
R15806			no Cq	no Cq				
R15808			no Cq	no Cq	25.54	no Cq		
R15842			no Cq	no Cq	29.54	no Cq		
R15849			no Cq	no Cq	34.42	no Cq	31.16	no Cq
R15859			no Cq	no Cq	no Cq	no Cq	26.62	no Cq
R15818					28.19	no Cq	28.01	no Cq
R15823					no Cq	no Cq	no Cq	no Cq
R15747					no Cq	no Cq	no Cq	no Cq
R15776					no Cq	no Cq		
R15743					no Cq	no Cq	25.02	no Cq
R15923					no Cq	no Cq		
R15756					no Cq	no Cq		
R15760					no Cq	no Cq	no Cq	no Cq
R15741					no Cq	no Cq	27.16	no Cq
R15742					no Cq	no Cq	32.58	no Cq
R15785					no Cq	no Cq	no Cq	no Cq
R15938					no Cq	no Cq		
R15927					27.10	no Cq	no Cq	no Cq
R32864					27.00	no Cq		
R15821					no Cq	no Cq	no Cq	no Cq
R15827					no Cq	no Cq		
R15775					no Cq	no Cq	34.71	no Cq
R15758					no Cq	no Cq	26.55	no Cq
R15752					no Cq	no Cq	34.19	no Cq
R15770					no Cq	no Cq		
R15774					no Cq	no Cq		
R15761					no Cq	no Cq	25.28	no Cq
R15751					no Cq	no Cq	31.59	no Cq
R15759					no Cq	no Cq	26.95	no Cq
R15787					no Cq	no Cq	22.16	no Cq
R15755					no Cq	no Cq	31.14	no Cq
R15745					no Cq	no Cq	34.57	no Cq
R15768					no Cq	no Cq	no Cq	no Cq
R15773					no Cq	no Cq	25.15	no Cq
R15828					no Cq	no Cq	36.27	no Cq
R15757					no Cq	no Cq	no Cq	no Cq
R15826					32.44	no Cq	no Cq	no Cq
R15820					29.30	no Cq	37.59	no Cq

R15814					26.32	no Cq	28.35	no Cq
R15779					no Cq	no Cq	no Cq	no Cq
R15816					no Cq	no Cq		
R02645					no Cq	no Cq		
R15783					28.23	no Cq	no Cq	no Cq
R15929					no Cq	no Cq	no Cq	no Cq
R15924					no Cq	no Cq	no Cq	no Cq
R15811					no Cq	no Cq	30.71	no Cq
R32901					no Cq	no Cq		
R15830					no Cq	no Cq	34.58	no Cq
R15767					no Cq	no Cq	no Cq	no Cq
R15790					no Cq	no Cq	30.92	no Cq
R15777					no Cq	no Cq	26.96	no Cq
R15772					no Cq	no Cq	no Cq	no Cq
R15769					no Cq	no Cq	30.58	no Cq
R15930					no Cq	no Cq		
R15788					no Cq	no Cq	no Cq	no Cq
R15813					no Cq	no Cq	no Cq	no Cq
R15786					no Cq	no Cq	29.16	no Cq
R15824					no Cq	no Cq		
R15817					23.79	no Cq		
R15928					no Cq	no Cq	no Cq	no Cq
R15822					no Cq	no Cq	no Cq	no Cq
R02430					no Cq	no Cq		
R15789					no Cq	no Cq		
R15825					no Cq	no Cq	27.04	no Cq
R15819					no Cq	no Cq	25.09	no Cq
R15922					26.45	no Cq	36.31	no Cq
R15780					no Cq	no Cq	24.74	no Cq
R15925					no Cq	no Cq	no Cq	no Cq
R15753					no Cq	no Cq	no Cq	no Cq
R15829					no Cq	no Cq	28.33	no Cq
R42432							no Cq	no Cq
R42441							no Cq	no Cq
R42447							no Cq	no Cq
R42453							29.28	no Cq
R42455							no Cq	no Cq
R15713							no Cq	no Cq
R15714							no Cq	no Cq
R15716							no Cq	no Cq
R15717							no Cq	no Cq
R15720							no Cq	no Cq
R15723							27.79	no Cq
R15724							no Cq	no Cq
R15725							no Cq	no Cq
R15730							no Cq	no Cq
R15731							20.72	no Cq
R15732							no Cq	no Cq
R15733							24.91	no Cq
R15734							no Cq	no Cq
R15735							31	no Cq
R15736							no Cq	no Cq
R15749							28.66	no Cq
R15750							no Cq	no Cq

R15766								29.83	no Cq
R83214								27.22	no Cq
R83252								33.61	no Cq
R39513								no Cq	no Cq
R39514								no Cq	no Cq
R39515								28.17	no Cq
R39516								no Cq	no Cq
R39517								no Cq	no Cq
R39518								no Cq	no Cq
R39519								no Cq	no Cq
R39520								33.02	no Cq
R39521								no Cq	no Cq
R39522								no Cq	no Cq
R39524								no Cq	no Cq
R39525								no Cq	no Cq
R39526								29.3	no Cq
R39527								28.14	no Cq
R39528								no Cq	no Cq
R39529								no Cq	no Cq
R39530								no Cq	no Cq
R39532								no Cq	no Cq
R39533								no Cq	no Cq
R39534								no Cq	no Cq
R39535								no Cq	no Cq
R39536								no Cq	no Cq
R39537								no Cq	no Cq
R39538								no Cq	no Cq
R39539								no Cq	no Cq
R39540								no Cq	no Cq
R39541								no Cq	no Cq
R39542								no Cq	no Cq
R39544								no Cq	no Cq
R39545								no Cq	no Cq
R39546								no Cq	no Cq
R39547								no Cq	no Cq
R39548								no Cq	no Cq
R39549								no Cq	no Cq
R39550								no Cq	no Cq
R39551								no Cq	no Cq
R39552								no Cq	no Cq
R39553								no Cq	no Cq
R39554								no Cq	no Cq
R39556								no Cq	no Cq
R39557								no Cq	no Cq
R39558								no Cq	no Cq
R39559								no Cq	no Cq
R39560								no Cq	no Cq
R39561								no Cq	no Cq
R39562								no Cq	no Cq
R39563								no Cq	no Cq
R39564								27.48	no Cq
R39565								no Cq	no Cq
R39566								no Cq	no Cq
R15763								no Cq	no Cq

Table S2: List of accession numbers and according sample ID for the sequences analysed in Figure 2

accession number	cattle number_sampling date_sample number	sequenced genome
OQ979546	R095_Oct20_BH60/20-50	partial NS3 gene
OQ979547	R197_Oct20_BH60/20-66	partial NS3 gene
OQ979548	R293_Oct22_BH33/22-145	partial NS3 gene
OQ979549	R297_Oct22_BH33/22-143	partial NS3 gene
OQ979550	R617_Oct20_BH60/20-40	partial NS3 gene
OQ979551	R689_Dec20_BH63/20-65	partial NS3 gene
OQ979552	R689_Oct22_BH33/22-229	partial NS3 gene
OQ979553	R732_Oct20_BH60/20-90	partial NS3 gene
OQ979554	R755_Dec20_BH63/20-69	partial NS3 gene
OQ979555	R755_Oct20_BH60/20-10	partial NS3 gene
OQ979556	R773_Dec20_BH63/20-08	partial NS3 gene
OQ979557	R773_Oct20_BH60/20-197	partial NS3 gene
OQ979558	R808_Oct22_BH33/22-55	partial NS3 gene
OQ979559	R814_Oct22_BH33/22-109	partial NS3 gene
OQ979560	R817_Oct22_BH33/22-204	partial NS3 gene
OQ979561	R839_Oct22_BH33/22-185	partial NS3 gene
OQ979562	R844_Jan22_BH02/22-04	partial NS3 gene
OQ979563	R844_Oct22_BH33/22-150	partial NS3 gene
OQ979564	R857_Dec20_BH63/20-39	partial NS3 gene
OQ979565	R857_Oct20_BH60/20-110	partial NS3 gene
OQ979566	R859_Dec20_BH63/20-44	partial NS3 gene
OQ979567	R859_Oct20_BH60/20-196	partial NS3 gene
OQ979568	R859_Sep21_BH54/21-39	partial NS3 gene
OQ979569	R862_Oct22_BH33/22-95	partial NS3 gene
OQ979570	R869_Dec20_BH63/20-85	partial NS3 gene
OQ979571	R869_Oct20_BH60/20-37	partial NS3 gene
OQ979572	R869_Oct22_BH33/22-173	partial NS3 gene
OQ979573	R869_Sep21_BH54/21-144	partial NS3 gene
OQ979574	R880_Dec20_BH63/20-05	partial NS3 gene
OQ979575	R880_Oct20_BH60/20-38	partial NS3 gene
OQ979576	R881_Dec20_BH63/20-76	partial NS3 gene
OQ979577	R881_Oct20_BH60/20-67	partial NS3 gene
OQ979578	R885_Dec20_BH63/20-40	partial NS3 gene
OQ979579	R885_Oct20_BH60/20-125	partial NS3 gene
OQ979580	R885_Oct22_BH33/22-221	partial NS3 gene
OQ979581	R885_Sep21_BH54/21-107	partial NS3 gene
OQ979582	R888_Dec20_BH63/20-36	partial NS3 gene
OQ979583	R888_Oct22_BH33/22-206	partial NS3 gene
OQ979584	R888_Oct20_BH60/20-03	partial NS3 gene
OQ979585	R905_Jan22_BH02/22-06	partial NS3 gene
OQ979586	R905_Sep21_BH54/21-49	partial NS3 gene
OQ979587	R922_Oct22_BH33/22-228	partial NS3 gene
OQ979588	R929_Oct20_BH60/20-108	partial NS3 gene
OQ979589	R935_Oct20_BH60/20-173	partial NS3 gene
OQ979590	R947_Dec20_BH63/20-80	partial NS3 gene
OQ979591	R947_Oct20_BH60/20-39	partial NS3 gene
OQ979592	R961_Dec20_BH63/20-20	partial NS3 gene
OQ979593	R961_Oct20_BH60/20-89	partial NS3 gene
OQ979594	R961_Oct22_BH33/22-128	partial NS3 gene
OQ979595	R965_Sep21_BH54/21-65	partial NS3 gene
OQ979596	R968_Oct22_BH33/22-11	partial NS3 gene
OQ979597	R997_Jan22_BH02/22-10	partial NS3 gene
OQ979598	R997_Oct22_BH33/22-219	partial NS3 gene
OQ979599	R997_Sep21_BH54/21-106	partial NS3 gene
OQ979600	R2869_Sep21_BH54/21-162	partial NS3 gene
